# GTPase regulator associated with the focal adhesion kinase (*GRAF*) transcript was down-regulated in patients with myeloid malignancies

**DOI:** 10.1186/1756-9966-29-111

**Published:** 2010-08-12

**Authors:** Zhen Qian, Jun Qian, Jiang Lin, Dong-ming Yao, Qin Chen, Run-bi Ji, Yun Li, Gao-fei Xiao, Jian-yong Li

**Affiliations:** 1 Department of Hematology, Affiliated People's Hospital of Jiangsu University, Zhenjiang, Jiangsu 212002, China; 2 Department of Hematology, The First Hospital of Nanjing Medical University, Jiangsu Provincial People's Hospital, Nanjing, Jiangsu, 210029, China

## Abstract

**Background:**

GTPase regulator associated with the focal adhesion kinase (*GRAF*), a putative tumor suppressor gene, is found inactivated in hematopoietic malignancies by either genetic or epigenetic abnormalities. However, the expression level of *GRAF *gene has not yet been studied in leukemia. The aim of this study was to investigate the expression level of *GRAF *gene in those patients with myeloid malignancies including acute myeloid leukemia (AML), myelodysplastic syndrome (MDS) and chronic myeloid leukemia (CML).

**Methods:**

The expression levels of *GRAF *transcript were determined in 94 patients using real-time quantitative PCR (RQ-PCR). Clinical and laboratory data of these patients were collected and analyzed.

**Results:**

The significantly decreased level of *GRAF *transcript was observed in three myeloid malignancies compared to controls. Within AML, there was no difference in the level of *GRAF *transcript among different FAB subtypes (*P *> 0.05). Difference was not observed in the amount of *GRAF *mRNA between CML at chronic phase and controls. As CML progressed, *GRAF *transcript significantly decreased. In MDS, three cases with 5q deletion had lower *GRAF *transcript than four without 5q deletion (median 0.76 vs 2.99) (*P *> 0.05).

**Conclusion:**

our results demonstrate that the *GRAF *transcript is decreased in myeloid malignancies.

## Background

Focal adhesion kinase (*FAK*), a non-receptor tyrosine kinase that resides at the sites of integrin clustering [1], plays an important role in the modulation of cell growth, proliferation, survival and migration [2]. Recently, *FAK *has been found to be overexpressed and/or constitutively activated and correlated with increased motility, invasiveness, and proliferation of neoplastic cells of various tissue types [2]. Two published articles revealed that aberrant expression of *FAK *was observed in CD34+ leukemic cells and associated with enhanced blast migration, increased cellularity and poor prognosis [3,4]. Le et al showed that *FAK *silencing inhibited leukemogenesis in *BCR/ABL*-transformed hematopoietic cells [5]. Tyner et al also identified *FAK *as one of therapeutic molecular targets in acute myeloid leukemia (AML) [6].

FAK protein is composed of an N-terminal FERM domain, a central kinase domain, and a C-terminal domain that includes the focal adhesion targeting (FAT) sequence responsible for FAK's localization to focal adhesions. Both the N-terminal and C-terminal domains have been shown to mediate FAK interaction with a variety of other proteins critical for activation of FAK by integrins or other cell surface receptors as well as FAK regulation of different cellular functions [2].

GTPase regulator associated with focal adhesion kinase (GRAF) is a newly identified protein specifically binding to the proline-rich region in the COOH terminus of FAK and negatively regulates the small GTP-binding protein RhoA, which is well known for its growth-promoting effect in RAS-mediated malignant transformation [7,8]. *GRAF *gene is located at chromosome 5q31 and its protein is ubiquitously expressed in various tissues [9]. Mutations and deletions of *GRAF *gene were found in some cases with AML or myelodysplastic syndrome (MDS) with a deletion 5q [9]. Furthermore, Bojesen et al [10] found that *GRAF *gene promoter was methylated in AML and MDS. The suppressed *GRAF *expression could be restored in leukemic cell lines by treatment with a demethyating agent and an inhibitor of histone deacytylases. However, the expression level of *GRAF *gene has not yet been studied in leukemia. We established the real-time quantitative polymerase chain reaction (RQ-PCR) assay with EvaGreen dye and examined the expression level of *GRAF *mRNA in myeloid malignancies.

## Materials and methods

### Patients and samples

The bone marrow mononuclear cells (BMNCs) from 94 patients with myeloid malignancies, including 72 AML, 7 MDS and 15 chronic myeloid leukemia (CML), were studied. The diagnosis and classification of AML and MDS patients were based on the French-American-British (FAB) and World Health Organization (WHO) criteria (blast ≥ 20%) combined to immunophenotyping and cytogenetic analysis [11-15]: among AML, 12 cases of M1, 23 cases of M2, 13 cases of M3, 18 cases of M4, 5 cases of M5, 1 case of M6; among MDS, 1 case of refractory anemia with ring sideroblasts (RARS), 2 cases of refractory cytopenia with multilineage dysplasia (RCMD), 3 cases of refractory anemia with excess blasts-1 (RAEB-1), 1 case of RAEB-2. The diagnosis of CML was established according to the conventional criteria [16]: 10 cases at chronic phase (CP), 5 cases at blast crisis (BC).

The clinical characteristics of patients were listed in Table 1. Karyotypes were analyzed using conventional R-banding method. Karyotype risk in AML and MDS was classified according to the reported studies [15,17]. t(15;17) was also included in the group of low risk. BMNCs, collected from 3 donors of bone marrow transplantation, 5 patients with immune thrombocytopenia (ITP), and 13 with iron deficiency anemia (IDA), were used as controls.

**Table 1:** clinical and laboratory features of patients with myeloid malignancies

Parameter	AML	CML	MDS
Age, median (range) (years)^a^	54(2-86)	52(11-75)	63(39-85)
Sex (male/female)	44/28	8/7	5/2
WBC (×10^9^/l)^a^	7.5(0.3-203.6)	83.4(2.8-168.7)	3.6(1.6-12.2)
Haemoglobin (g/dl)^a^	71(24-123)	91(50-134)	64(46-91)
Platelet count (×10^9^/l)^a^	40(3-447)	200(20-850)	50(10-926)
Cytogenetics			
Good	22		3
Intermediate	35		3
Poor	8		1
CD34(+/-)	35/26		
*GRAF *level^a^	3.88(0.01-169.75)^b^	23.51(0.01-157.42)^c^	10.20(0.25-45.90)^b^

WBC, white blood cells; ^a^Median (range); ^b^*P *< 0.001, compared with control; ^c^*P *= 0.030, compared with control;

### Immunophenotyping studies

Erythrocyte-lysed whole BM samples from 61 AML patients were analyzed by flow cytometry using a panel of MoAbs in triple stainings [phycoerythrin (PE)/fluorescein isothiocyanate (FITC)/Peridin chlorophyll (PerCP)]: IgG1-FITC; IgG2a-PE; CD2-PE; CD4-FITC; CD7-FITC; CD10-FITC; CD11b-PE; CD13-PE; CD19-PE; CD38-PE; CD45-PerCP; CD117-PE; HLA-DR-FITC (Becton Dickinson, USA); CD14-PE; CD22-FITC; CD33-FITC; CD34-FITC; CD36-FITC (Beckman Coulter, USA). Data acquisition and analysis were performed on a FACScalibur flow cytometer (Becton Dickinson) using Cell-Quest software. Identification of leukemic cells was performed using CD45 intensity versus SSC dot plots. Antigen expression was considered to be positive when the percentage of positive leukemic cells was equal or greater than 20%.

### Preparation of RNA and cDNA synthesis

BMNCs were separated using Lymphoprep and lysed with Trizol (In Vitrogen, Carlsbad, CA, USA) according to the manufacturer's instructions. Two micrograms of total RNA was reverse transcribed to cDNA in a total reaction volume of 40 μl containing 5× buffer, dNTPs 10 mM each, random hexamers 10 μM, RNAsin 80 units and 200 units of MMLV reverse transcriptase (MBI Fermentas, USA). Samples were incubated for 10 min at 25°C, 60 min at 42°C, and then stored at -20°C.

### RQ-PCR

RQ-PCR was performed using EvaGreen dye (BIOTIUM, Hayward, CA, USA) on a 7300 Thermo cycler (Applied Biosystems, Foster City, CA, USA). Real-time fluorescent data were collected and analyzed with SDS 1.3 software (Applied Biosystems, Foster City, CA, USA). The baseline fluorescence intensities were fixed at cycles 6-15 by default and 0.01 was set as the threshold to determine the cycle threshold (C_T_) value. The primers of *GRAF *and housekeeping gene *ABL *were designed against GenBank-published sequences (NM_015071 and NM_14752) with the software Primer Express 2.0 (Applied Biosystems, Foster City, CA, USA). The primer sequences are as follows: *GRAF *forward 5'-ATTCCAGCAGCAGCTTACA-3', reverse 5'-GATGAGGTGGGCA TAGGG-3', *ABL *forward 5'-TCCTCCAGCTGTTATCTGGAAGA-3', reverse 5'-TCCAACGA GCGGCTTCAC-3', with expected PCR products of 166 bp and 118 bp, respectively. PCR was performed in a final volume of 25 μl, containing 100 ng of cDNA, 0.2 mM of dNTP, 4 mM of MgCl_2_, 0.4 μM of primers, 1.2 μl of EvaGreen, 1.0 U of Taq DNA Polymerase (MBI Fermentas, USA). Amplification consisted of an initial denaturation step of 94°C for 4 min followed by 40 cycles of a denaturation step at 94°C for 30 s, an annealing step at 62°C for 30 s, an extension step of 72°C for 30 s, and an fluorescence collection step at 82°C for 30 s, followed by a final extension of 72°C for 10 min. Sterile H2O without cDNA used as no-template control (NTC) in each assay. The copies of *GRAF *and *ABL *mRNA were calculated automatically by the software. The relative amount of *GRAF *was normalized using the following formula: N_*GRAF *_= (copies of *GRAF*/copies of *ABL*) × 100. Amplified RQ-PCR products from three samples were sequenced (Shanghai GeneCore BioTechnologies Co., Ltd., China).

### Statistical analyses

Statistics was performed using the SPSS 13.0 software package (SPSS, Chicago, IL). The Kruskal-Wallis test (multiple groups) and Mann-Whitney *U*-test (two groups) were employed to compare the difference between patient groups and controls. The correlation between the level of *GRAF *transcript and the sex, age, hematologic parameters, FAB subtypes and karyotypic groups was calculated by Spearman's rho correlation analyses. A *P*-value < 0.05 was considered significant.

## Results

### GRAF expression in controls and AML patients

**Figure 1:**
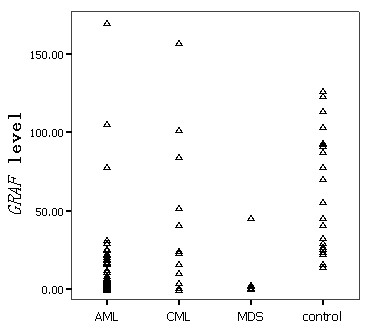
**Scatterplot showing varying levels of *GRAF *transcript in patients with different myeloid malignancies and controls**.

The level of *GRAF *transcript in controls was 14.49-126.85 (median 56.04). The significantly decreased level of *GRAF *transcript was observed in different myeloid malignancies (Table 1, Figure 1). There was no correlation between *GRAF *mRNA amount and the sex, age, peripheral white blood cell count, hemoglobin level, and platelet count (*P *> 0.05). The association of *GRAF *levels with cytogenetic abnormalities or CD34 antigen expression was also not observed in AML patients (*P *> 0.05). Within AML, there was no difference in the level of *GRAF *transcript among different FAB subtypes (*P *> 0.05).

### GRAF expression in CML patients

**Figure 2:**
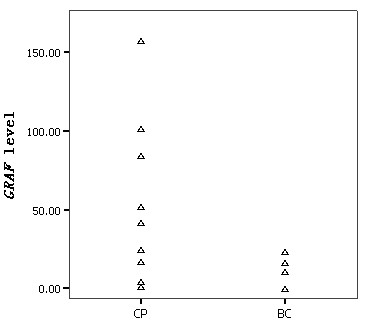
**Expression level of *GRAF *transcript in CML**.

The median levels of *GRAF *transcript in CML patients at CP and BC were 46.82 (1.08-157.42) and 10.69 (0.01-23.51), respectively (Figure 2). There was no difference in *GRAF *transcript amount between CML patients at CP and controls (*P *> 0.05). However, the amount of *GRAF *mRNA in CML at BC was significantly lower than that in cases at CP and that in controls (*P *= 0.028 and <0.001, respectively).

### GRAF expression in MDS patients

Among MDS patients, three cases were identified with deletions of 5q (5q-) (Table 2). The level of *GRAF *transcript was lower in these cases (0.49-1.02, median 0.76) than the other four cases without 5q- (0.25-45.90, median 2.99), however, statistical difference was not observed (*P *> 0.05).

**Table 2:** Clinical and laboratory characteristics of patients with MDS

No.	Sex	Age (year)	Diagnosis	Karyotype	*GRAF *level
1	F	51	RAEB-2	46, XX	2.76
2	F	63	RCMD	46, XX, del(20)(q11)	45.90
3	M	67	RAEB-1	46, XY	3.22
4	M	74	RARS	46, XY, del(5)(q13q33)	0.49
5	M	85	RAEB-1	46, XY, del(5)(q13q33)	0.76
6	M	39	RCMD	46, XY	0.25
7	M	41	RAEB-1	44-45, XY, del(5)(q13q33), -7, -15, -21[cp]	1.02

## Discussion

In this study, we demonstrated that the expression level of *GRAF *transcript was decreased in primary leukemic cells of all types of myeloid malignancies. Bojesen et al [10] found that *GRAF *promoter was hypermethylated in 38% cases with AML and MDS but not in healthy individuals, however, they did not detect the *GRAF *transcript in primary leukemic cells of AML and MDS. GRAF contains a centrally located GTPase-activating protein (GAP) domain, followed by a serine/proline rich domain and a carboxy-terminal Srchomology 3 (SH3) domain. GRAF acts as a negative regulator of RhoA because the GRAF GAP domain enhances GTP hydrolysis of both Cdc42 and RhoA in vitro [7]. Rho family GTPases play a role in the growth control besides regulating the organization of the actin cytoskeleton [18,19]. RhoA inhibits p21Cip1, p27Kip and p16Ink4 activities, permitting cell cycle progression [20-24]. Furthermore, RhoA has been shown involved in the regulation of apoptosis, migration, proliferation, differentiation [18,19]: for example, in vitro, constitutively active RhoA can stimulate transformation. In normal epithelia, RhoA contributes to the generation of epithelial polarity and junction assembly and function but also affects epithelial disruption during tumor progression [25]. Recently, clinical studies have revealed the correlation of increased expression of RhoA and invasion, metastasis and progression of several solid tumors including liver, bladder, esophageal, head and neck, ovary, gastric, testicular, lung and breast carcinomas [18]. As an upstream regulator, the loss of function of *GRAF *might prevent the physiologic down-regulation of RhoA and lead to the repression of p21. Then, the *GRAF*-defective cell will be driven into the S phase [9]. Several mechanisms, including translocations, allelic loss, insertions and promoter methylation observed in AML and MDS, can lead to the inactivation of *GRAF *[9,10].

The mechanisms responsible for the disease progression of CML remained poorly understood. Recent studies have suggested that several alterations promote this progress, including differentiation arrest caused by the suppression of translation of the transcription factor CEBPα induced by the BCR-ABL oncoprotein in CML cell, increasing genomic instability in CML cell resulting from the reduced capability of genome surveillance system, telomere shortening and loss of tumor suppressor gene (TSG) such as *TP53*, retinoblastoma 1, *CDKN2A*, *DAPK1 *and others [16,26,27]. Interestingly, we found that *GRAF *transcript was further down-regulated during CML progression. p210 Bcr-Abl, containing a centrally located Rho-specific guanine nucleotide exchange factors (RhoGEF) domain, affects the actin cytoskeleton assembly and thereby the cellular adhesion and migration by RhoA signaling pathway [28]. Further studies are required to elucidate the function of *GRAF *and *RhoA *in the pathogenesis and progression of CML.

Our preliminary results showed that MDS with 5q deletion might have lower expression of *GRAF *than those without 5q deletion. Deleted 5q is a one of common chromosomal abnormalities in AML and MDS. Although *GRAF *maps telomeric to the previously delineated commonly deleted 5(q31) region, Borkhardt et al found that one allele of *GRAF *was consistently lost in all studied 10 patients with 5q deletion and with either MDS or AML [9]. Besides *GRAF *deletion, abnormal methylation of *GRAF *promoter was also observed in AML and MDS [10]. These results suggested that haploinsufficiency (i.e., decreased *GRAF *mRNA expression) caused by deletion of *GRAF *allele or promoter methylation might be instrumental in the development and progression of hematopoietic malignancies.

In conclusion, *GRAF *mRNA is decreased in myeloid malignancies. Whether the *GRAF *expression level could improve the stratification or prognostication of patients with myeloid diseases should be further addressed in future studies.

## Competing interests

The authors declare that they have no competing interests.

## Authors' contributions

QJ and LJY designed the study, analyzed the data and wrote the manuscript; QZ, LJ, YDM and CQ performed all experiments; JRB, LY and XGF gave assistance with technical performance and contributed to the writing of the manuscript. All authors read and approved the final manuscript.
